# Deviating HER2 test results in gastric cancer: analysis from the prospective multicenter VARIANZ study

**DOI:** 10.1007/s00432-022-04208-6

**Published:** 2022-08-27

**Authors:** Katharina Kolbe, Ivonne Haffner, Katrin Schierle, Dieter Maier, Birgitta Geier, Birgit Luber, Hendrik Bläker, Christian Wittekind, Florian Lordick

**Affiliations:** 1 Department of Oncology, Gastroenterology, Hepatology, Pulmonology and Infectious Disease, Leipzig University Medical Center, University Cancer Center Leipzig (UCCL), Leipzig, Germany; 2 Institute of Pathology, Heilbronn SLK-Kliniken GmbH, Heilbronn, Germany; 3 Biomax Informatics AG, 82152 Planegg, Germany; 4 Institute of Pathology, Technische Universität München, Munich, Germany; 5 Department of Pathology, Leipzig University Medical Center, Leipzig, Germany

**Keywords:** Gastric cancer, Esophago-gastric junction cancer, HER2 status, Trastuzumab, Survival, Immunohistochemistry

## Abstract

**Purpose:**

The prospective multicenter VARIANZ study aimed to identify resistance biomarkers for HER2-targeted treatment in advanced gastric and esophago-gastric junction cancer (GC, EGJC). HER2 test deviations were found in 90 (22.3%) of 404 cases (central versus local testing) and were associated with negative impact on survival for trastuzumab-treated patients. Here, we investigated methodological and biological variables that may promote deviating HER2 test results.

**Methods:**

We analyzed HER2 testing procedures and participation in quality assurance programs of 105 participating local pathology laboratories. Furthermore, tumor localization and histological subtypes were compared between patients with centrally confirmed (central HER2 + /local HER2 + , *n* = 68) and unconfirmed HER2 status (central HER2 −/local HER2 + , *n* = 68).

**Results:**

For central HER2 testing, concordance between in situ hybridization (ISH) and immunohistochemistry (IHC) was 98.3%, with IHC sensitivity of 93.3% (84 IHC + of 90 ISH +), specificity of 99.5% (389 IHC- of 391 ISH-), and a positive diagnosis rate of 97.7%. Central confirmation of the local HER2 IHC scores were seen for the majority of locally HER2- IHC 0/1 (172/178; 96.6%), but less frequently for locally IHC3 + (57/124; 46.0%) cases. Deviation rate was not associated with IHC antibody platform used in the local pathology institute neither with participation in quality-assuring tests. Regarding tumor characteristics, deviating test results were more frequently found in GC vs. EGJC (69.1% vs. 39.7%; *p* = 0.001) and in Laurén diffuse vs. intestinal subtype (23.5% vs. 5.9%, *p* = 0.004).

**Conclusion:**

Tumor localization and histological subtype have an impact on HER2 test deviation rates. Assessment of HER2 remains challenging for GC and EGJC.

**Supplementary Information:**

The online version contains supplementary material available at 10.1007/s00432-022-04208-6.

## Introduction

Globally, gastric cancer (GC) is the fifth most common cancer and the third leading cause of cancer-related deaths (Siegel et al. 2018). In the Western hemisphere, the majority of patients are diagnosed at advanced stages and median overall survival (mOS) is less than 1 year when patients are treated with platinum–fluoropyrimidine combination chemotherapy (Smyth et al. 2020). The Trastuzumab for Gastric Cancer (ToGA) study established the value of trastuzumab, an anti-HER2-directed monoclonal antibody, for treatment of HER2 + GC and esophago-gastric junction (EGJC). Until today, trastuzumab is the only targeted drug that has shown to improve OS of HER2-positive (HER2 +) GC patients in a randomized controlled phase III trial (Bang et al. [Bibr CR3][Bibr CR3], b). In contrast, some other HER2-targeting drugs have failed to improve survival outcomes in phase III trials (Hecht et al. 2016; Satoh et al. 2014; Tabernero et al. 2018; Thuss-Patience et al. 2017), while novel promising compounds are currently being studied (Bang et al. 2017; Kim et al. 2019; Shitara et al. 2020). HER2 overexpression and/or amplification is reported in 10–27% of the patients with advanced GC (Chua and Merrett 2012; Koopman et al. 2015; Press et al. 2017; Yoon et al. 2012). GC-specific guidelines for HER2 testing have been developed (Rüschoff et al. 2010). In contrast to breast cancer, basolateral HER2 staining in immunohistochemistry (IHC) is considered HER2 + because incomplete membranous staining is common in GC (Hofmann et al. 2008; Rüschoff et al. 2012). Furthermore, GC often shows heterogeneous HER2 expression (Grabsch et al. 2010; Leni et al. 2014; Peng et al. 2015; van Cutsem et al. 2015; Yang et al. 2012), which makes diagnostics even more complicated.

According to current recommendations (Bang et al. 2010a, b; Rüschoff et al. 2010), HER2 is primarily analyzed by IHC. Various antibodies from different commercial providers are available for HER2 IHC testing. For ToGA and other studies, the HercepTest (Dako, Glostrup, Denmark) was used (Dijksterhuis et al. 2020; He et al. 2015; Yoon et al. 2012), while other studies preferred the 4B5 HER2-antibody (Ventana Medical Systems, Tucson, AZ, United States). Concordance between the available antibodies has been demonstrated previously (Asioli et al. 2012; Boers et al. 2011; Cho et al. 2013; Radu et al. 2012; Rüschoff et al. 2010). However, 4B5 has also been reported of being more sensitive, leading to stronger HER2 membrane staining, but also non-specific cytoplasmic staining (Abrahão-Machado et al. 2013; Boers et al. 2011). CB11 (DCS, Hamburg, Germany) is a so far rarely used antibody (Grabsch et al. 2010) that is described as particularly specific, but less sensitive than other clones (Cappellesso et al. 2015; Cho et al. 2012; Schrohl et al. 2011). Currently, no specific HER2 companion diagnostic antibody is recommended for routine use in Europe (Baretton et al. 2019).

Current reports indicate that tumor biopsies are more often tested HER2 + than surgical specimens (Baretton et al. 2019; Kaito et al. 2019), and concordance of HER2 positivity between matched biopsies and surgical specimens or metastatic samples has been demonstrated in 96.1% and 94.9%, respectively (Bozzetti et al. 2011; Wang et al. 2014). However, it is also known that deviating HER2 test results occur when one investigator assessed different biopsies or different parts of a surgical specimen in the same patient (Xu et al. 2019). Additional block analysis increased both the sensitivity (from 63 to 83%) and the accuracy (from 91 to 94%) of IHC as compared with fluorescent in situ hybridization (Asioli et al. 2012).

HER2 + status is more frequently found in intestinal type than in diffuse type GC according to Laurén’s classification (Baretton et al. 2019; Cappellesso et al. 2015; Cho et al. 2013; Chua and Merrett 2012; Gomez-Martin et al. 2013; Gómez-Martin et al. 2012; Grabsch et al. 2010). EGJC tend to be more often HER2 + (24–33%) compared to distal GC (7–21%) (Baretton et al. 2019; Boers et al. 2011; van Cutsem et al. 2015; Yoon et al. 2012) which might be related to a higher rate of intestinal phenotype within EGJC (Shah et al. 2011). However, not all patients with HER2 + GC/EGJC seem to benefit from trastuzumab (Fu et al. 2018; Gomez-Martin et al. 2013; Lordick and Janjigian 2016; Yagi et al. 2019; Yi et al. 2016). The academic network study VARIANZ aimed to discover biomarkers to predict response or resistance to treatment with trastuzumab (Haffner et al. 2021). The majority of patients in VARIANZ were tested twice for tumor HER2 expression: once in local pathology institutes for diagnostic reasons and a second time in the central academic pathology institute as a study procedure. In 22.3% of the cases, central HER2 test results deviated from local tests. A significant survival advantage for patients treated with trastuzumab and a centrally confirmed HER2 + status (HER2 + /HER2 +) compared to a non-confirmed result.

(HER2 −/HER2 +) was reported previously (Haffner et al. 2021). In the present secondary analysis, we aimed to investigate whether deviating HER2 test results might be associated with the HER2 testing methodology or with tumor characteristics.

## Methods

### Study design

VARIANZ is a prospective, non-interventional multicenter study which recruited 548 patients at 35 German sites from 2014 to 2018. VARIANZ was funded by the German Federal Ministry of Education and Research (grant number: BMBF 01ZX1610E). Main results were published previously (Haffner et al. 2021). Briefly, adult patients receiving the first-line chemotherapy with or without trastuzumab for histologically confirmed advanced GC or EGJC were recruited. The primary endpoint was survival (OS), defined as time from the beginning of first-line chemotherapy until death from disease related cause. VARIANZ was conducted in accordance with Good Clinical Practice guidelines and the Declaration of Helsinki. All patients had given written informed consent. Approvals for the study protocol were obtained from ethics committees of all study sites.

### HER2 testing

The first HER2 diagnostic test was performed in local pathology institutes to determine the HER2 status and decide for the indication for trastuzumab therapy. The same or another tumor block was sent to the central pathology institute (Leipzig University Medical Center, Department of Pathology, Leipzig, Germany). HER2 expression was assessed centrally on fresh cut slides of the provided tumor block originating from primary tumor biopsies, surgical specimen or metastatic lesions using IHC (CB11 antibody, DCS, HI608C0I, Hamburg, Germany), and chromogenic in situ hybridization (ISH) (Zytomed Systems, C-3022-40, Berlin, Germany) according to established scoring criteria (Hofmann et al. 2008). The central laboratory did not receive the locally stained tissue sections. The IHC assessment was performed on fresh cut and newly stained slides without knowledge of the ISH results (and vice versa). ISH was performed and analyzed for all specimens regardless of the IHC test results. For IHC, all tumor cells per slide were analyzed and for ISH 20 cells were scored. For borderline cases (HER2/CEP17 ratio of 1.8–2.2), 40 more cells were analyzed. For HER2 evaluation, done by two dedicated GI pathologists at the central study pathology institute, no technical support or image analysis system was used.

In the country where this study was conducted, round robin tests of “Qualitätssicherungs-Initiative Pathologie QuIP GmbH” (https://www.quip.eu/de_DE/) are offered and recommended by the German Society of Pathology. For this purpose, QuIP sends unstained slides of gastric cancer to participating centers, on which either IHC or ISH or both are carried out. After evaluating the stained slides and corresponding results are sent back from the participating center to the QuIP. The QuIP then evaluates the section preparations with regard to staining quality and result accuracy.

### Survey on HER2 test methodology

Tumor specimens from 105 pathology institutes were sent to the central pathology. All participating pathologists were asked to complete a questionnaire containing information about the tumor tissue (biopsy versus resection specimen; primary tumor versus metastatic site), their participation in round robin tests, and the applied HER2 test method. We asked specifically for the following antibodies for IHC: HercepTest and A0485 (Dako, Glostrup, Denmark), SP3 (Labvision; Thermo Fisher Scientific, Fremont, CA, United States), 4B5 (Ventana Medical Systems, Tucson, AZ, United States), CB11 (Novocastra, Newcastle upon Tyne, Great Britain), UMAB36 (OriGene Technologies, Rockville, United States), and EP3 (Cell Marque, Rocklin, United States).

### Statistical methods

Stratification into groups was done according to central and local HER2 test results: HER2 +/HER2 + (central HER2 +/local HER2 +), HER2 +/HER2 − (central HER2 +/local HER2 −), HER2 −/HER2 + (central HER2 −/local HER2 +) and HER2 − /HER2 − (central HER2 −/local HER2 −). Comparative analysis such as sex, initial tumor stage, prior gastric surgery, tumor location, and histopathological subtype according to Laurén’s classification of the different patient cohorts (HER2 + /HER2 + vs. HER2 −/HER2 +) was carried out using the Chi^2^ test. *P* < 0.05 was considered statistically significant. Analyses were done with IBM SPSS version 24.0 (IBM Corp., Armonk, NY).

## Results

### Patient groups

**Fig.1:**
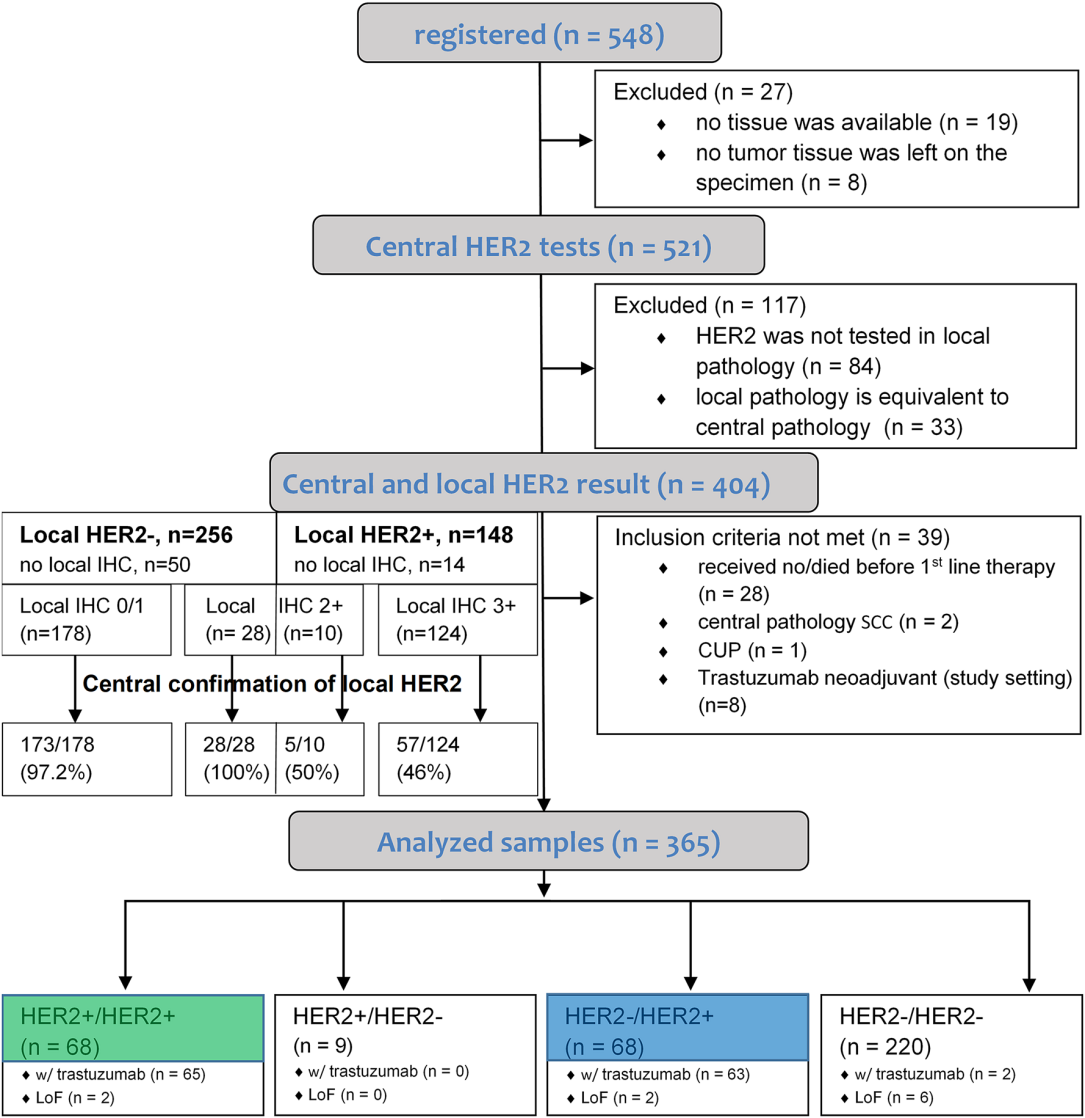
CONSORT diagram of the VARIANZ study. Patients were assigned to four groups according to confirmation of local HER2 status. *CUP* carcinoma of unknown primary, *HER2* human epidermal growth factor receptor 2, *LoF* lost to follow-up, *SC*C squamous cell carcinoma

Tumor tissue for central HER2 testing was available for 521 of 548 enrolled patients (Fig. 1). In 404 patients, HER2 status was determined in local and central pathology institutes. In local HER2 testing, 148 out of those 404 patients were rated HER2 positive. The central result differed in particular in the initially IHC3 + tested patients.

Of these 404 patients, 373 patients received first-line therapy and basis documentation was obtained. They were assigned to the four groups HER2 +/HER2 − (*n* = 9), HER2 + /HER2 + (*n* = 68; Fig. 1 green), HER2 −/HER2 + (*n* = 68; Fig. 1 blue), and HER2-/HER2- (*n* = 220).

### Tumor phenotype and localization

Diffuse subtype was less common in the HER2 + /HER2 + versus the HER2 −/HER2 + cohort [5.9% (*n* = 4) vs. 23.5% (*n* = 16); *p* = 0.004]. Laurén´s classification is unknown in a significant proportion of the confirmed and unconfirmed cohorts [39.0% (*n* = 53)]. Tumor localization was significantly different between the HER2 + /HER2 + and HER2 −/HER2 + cohorts (Table 1). More patients with EGJC were found in the HER2 + /HER2 + group [69.1% (*n* = 47)], while more GC patients were allocated in the HER2 −/HER2 + group [58.8% (*n* = 40); *p* = 0.001]. Centrally confirmed HER2 positivity rate was higher for EGJC (29.4%) and low for GC (10.8%).

**Table 1:** Characteristics of the HER2 + /HER2 + versus HER2-/HER2 + cohorts

	HER2 + /HER2 +	HER2-/HER2 +	Total	*p* Value (test)
	*n* = 68	*n* = 68	*n* = 136	
Age (mean ± SD)	65.5 ± 12.9	66.5 ± 10.2	66.0 ± 11.6	0.617 (*t* test)
Sex: male/female	53/15	57/11	110/26	0.383 (Chi^2^)
Prior gastric surgery	18 (26.5%)	21 (30.9%)	39 (28.7%)	0.569 (Chi^2^)
Stage IV at	52 (76.5%)	47 (58.8%)	99 (72.8%)	0.335 (Chi^2^)
initial diagnosis				
Tumor localization				
Non-cardia GC	21 (30.9%)	40 (58.8%)	61 (44.9%)	0.001 (Chi^2^)
EGJC	47 (69.1%)	28 (41.2%)	75 (55.1%)	0.001 (Chi^2^)
Laurén subtype				
Intestinal	30 (44.1%)	27 (39.7%)	57 (41.9%)	0.602 (Chi^2^)
Diffuse	4 (5.9%)	16 (23.5%)	20 (14.7%)	0.004 (Chi^2^)
Mixed type	2 (2.9%)	4 (5.9%)	6 (4.4%)	0.404 (Chi^2^)
No information	32 (47.1%)	21 (30.9%)	53 (39.0%)	0.053 (Chi^2^)
Local HER2 IHC result				
IHC2 +	3 (4.4%)	7 (10.3%)	10 (7.4%)	0.189 (Chi^2^)
IHC3 +	57 (83.8%)	54 (79.4%)	111 (81.6%)	0.507 (Chi^2^)
No information	8 (11.8%)	7 (10.3%)	15 (11.0%)	0.784
Central HER2 IHC result				
IHC0	0	29 (42.6%)	29 (21.3%)	
IHC1	0	14 (20.6%)	14 (10.3%)	
IHC2	13 (19.1%)	25 (36.8%)	38 (27.9%)	
IHC3	55 (80.9%)	0	55 (40.4%)	

There was no difference between the groups regarding local IHC testing (IHC2 + vs. IHC3 +).

Looking at the whole cohort (see Suppl. 1), there is a generally increased occurrence of EGJC in the HER2-positive cohort (*p* < 0.00001). With regard to the histological subtype, the assessment was not possible due to a high rate of missing information. However, there is the tendency of the increase in the intestinal and the decrease of the diffuse type in the HER2-positive cohort.

### HER2 test results

The following analysis of the test results refers to different sub-groups as detailed in the consort diagram (Fig. 1). For better comprehensibility, there is a modified scheme in the supplements (see Suppl. 2).

**Fig. 2:**
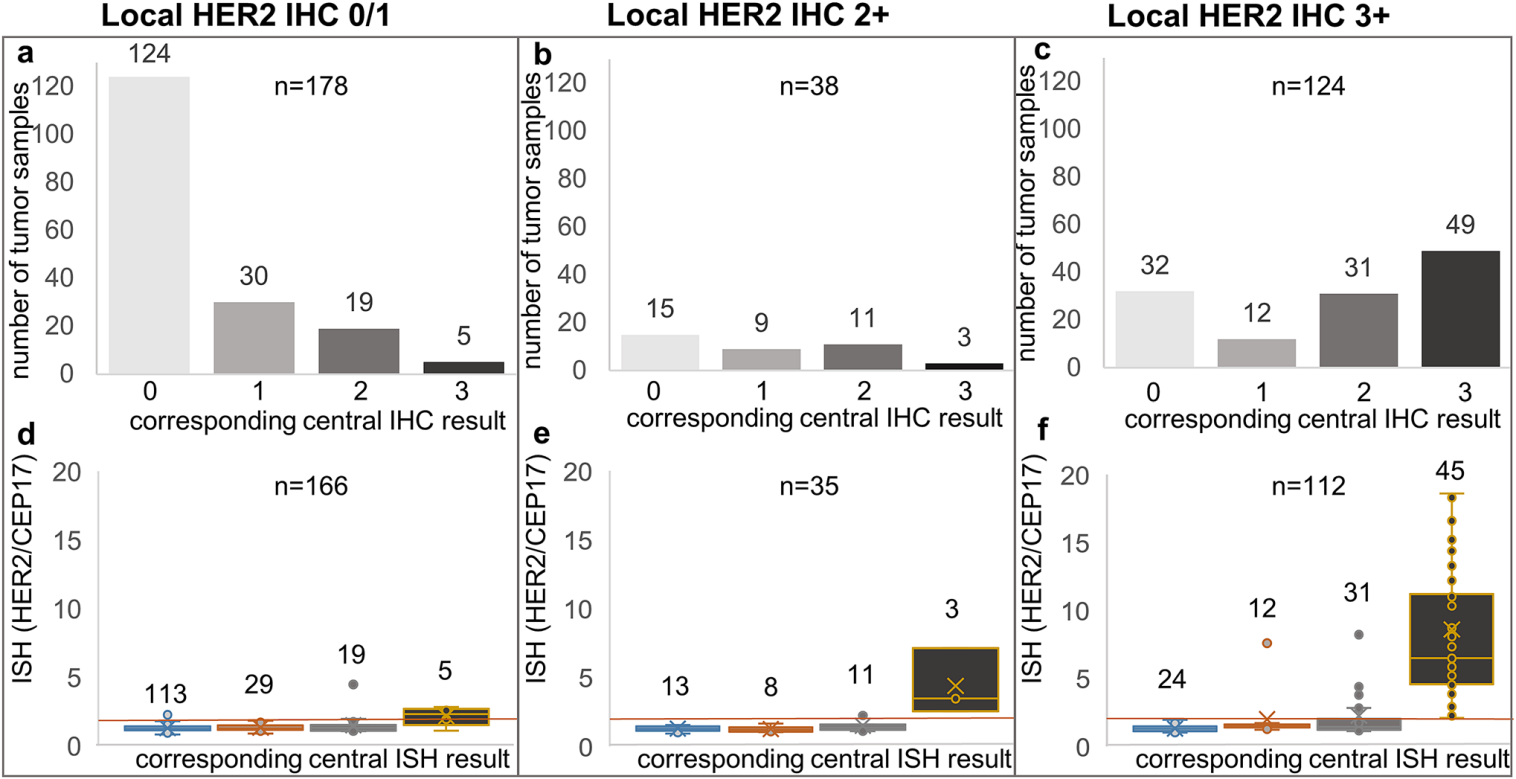
Comparison of local and central HER2 immunohistochemistry (IHC) scores and corresponding central in situ hybridization (ISH). HER2/CEP17 ratio ≥ 2 indicates HER2 positivity and is shown as orange line. **a** Tumor samples tested HER2 negative (IHC0/1) by local laboratories (*n* = 178) are shown. In these probes, central HER2 test was negative in 86.5% (IHC0 (*n* = 124); IHC1 (*n* = 30)); 10.7% were scored IHC2 + (*n* = 19) and 2.8% IHC3 + (*n* = 5). Corresponding central ISH results are shown in **d** with 5 of 166 tumors showing a HER2/CEP17 ratio ≥ 2. **b** Tumor samples with local IHC2 + tests (*n* = 38). Corresponding central IHC reveals an IHC score 0 in 39.5% (*n* = 15), IHC1 in 23.7% (*n* = 9), IHC2 in 28.9% (*n* = 11), and IHC3 in 7.9% (*n* = 3). Corresponding central ISH is shown in** e** with 5 of 35 samples showing an HER2/CEP17 ratio > 2. **c** Tumor samples with IHC3 + in the local laboratory (*n* = 124) are shown. Corresponding central IHC results are shown with negative HER2 in 35.5% (IHC0, *n* = 32 and IHC1, *n* = 12). 25.0% revealed intermediate HER2 expression (IHC2 + (*n* = 31)) of which 25.8% (*n* = 8) were confirmed HER2 + in central ISH (HER2/CEP17 ratio > 2). 39.5% confirmed IHC3 + (*n* = 49) in central IHC. Corresponding central ISH is shown in **f** with 57 of 112 samples showing a HER2/CEP17 ratio > 2

89 (17.1%) of 521 centrally analyzed tumor samples were HER2 + , with an IHC3 + in 70 patients and IHC2 + /ISH + in 19 patients. In 90 of the 404 (22.3%) patients with local and central HER2 test results, local HER2 status was not confirmed. The discordance of IHC results between central and local pathologies is shown in Fig. 2. The analysis included patients for whom a central test result and a concrete local IHC score were available. There was a particularly high discordance in samples scored HER2 IHC3 + locally (Fig. 2c, f) with only 57 samples (46.0%) confirming local HER2 + in the central laboratory.

Central HER2 ISH analysis was performed in 481 of 521 samples. Concordance between ISH and IHC results was 98.3% (473 of 481 tests). The eight discordant cases were two with positive HER2 IHC3 + but HER2/CEP17 ratio < 2.0 and six with HER2 IHC0/1 and HER2/CEP17 ratio ≥ 2.0 resulting in a sensitivity of 93.3% (84 IHC + of 90 ISH +) and a specificity of 99.5% (389 IHC- of 391 ISH-) and a positive diagnosis rate of 97.7%.

### HER2 test platforms

**Fig. 3:**
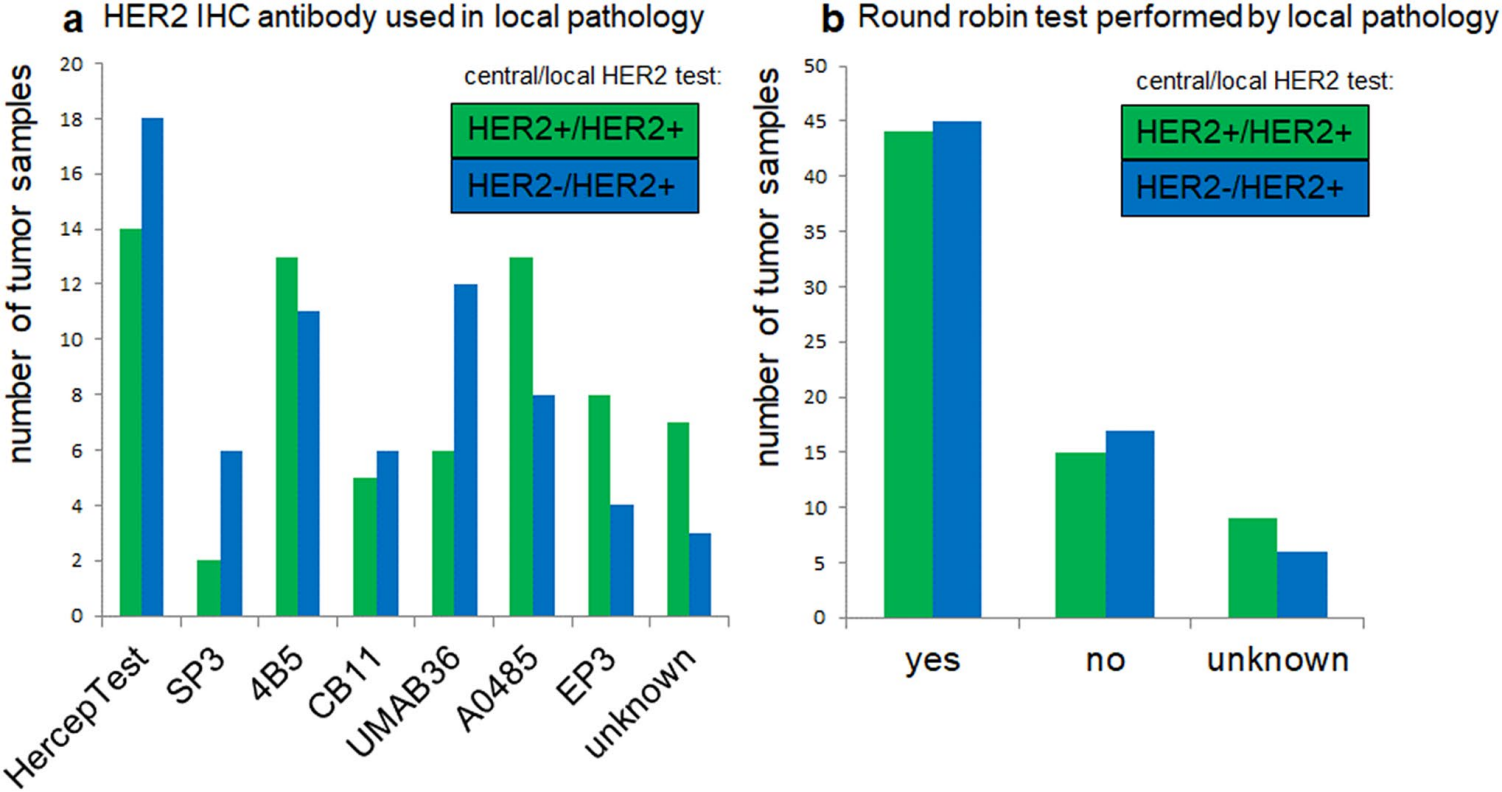
HER2 test platform in routine use. **a** Antibody used for local HER2 IHC, shown for the HER2 + /HER2 + (green, *n* = 68) and the HER2-/HER2 + (blue, *n* = 68) cohorts. **b** Participation in round robin tests for HER2 in GC, shown for the HER2 + /HER2 + (green, *n* = 68) and HER2-/HER2 + (blue, *n* = 68) cohorts; The Chi^2^ test did not show any significant differences

In local pathology laboratories, HercepTest [*n* = 23 (21.9%)] and 4B5 [*n* = 19 (18.1%)] were the most commonly used antibodies. Rarely applied antibodies were clones SP3 [*n* = 10 (9.5%)], CB11 [*n* = 6 (5.7%)], UMAB36 [*n* = 6 (5.7%)], A0485 [*n* = 11 (10.5%)], and EP3 [*n* = 6 (5.7%)]. 46 of 105 pathology laboratories (43.8%) participated in round robin tests, 25 (23.8%) did not participate, and for 34 (32.4%) participation is unknown despite repeated queries. No associations between HER2 test deviations and the applied IHC antibody (Fig. 3a) or participation in round robin tests on HER2 test deviations were demonstrated (Fig. 3b).

### Tumor material

**Fig. 4:**
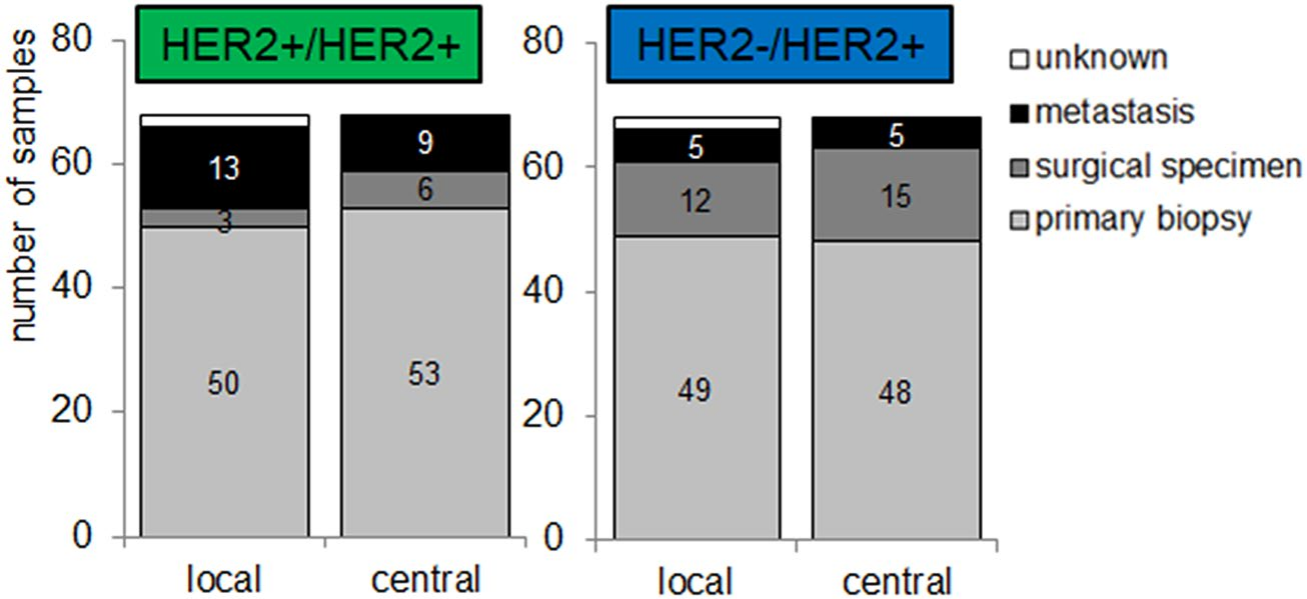
Tumor material used for HER2 assessment in local and central pathology laboratories, shown for the HER2 + /HER2 + (*n* = 68; left) and HER2-/HER2 + (*n* = 68; right) cohorts

The majority of tested tumor specimens was from primary biopsies (Fig. 4) followed by resection specimens or metastases, with no significant difference between the groups HER2 + /HER2 + and HER2 −/HER2 + . In 80.3% of the patients, tumor specimens originating from the same location (endoscopic biopsy of the primary tumor, or resection specimen, or biopsy from metastases) were tested for HER2 in both the central and local pathology laboratories (comparable cases, *n* = 390). For HER2 + /HER2 + and HER2-/HER2 + patients, this concordance of examined tumor specimens was comparable (83.8% vs. 85.3%, respectively).

## Discussion

The VARIANZ study has shown that GC patients with deviating HER2 test results had no benefit from HER2-targeted treatment with trastuzumab (Haffner et al. 2021). We wanted to understand the underlying causes for HER2 deviations. Our analyses demonstrates that neither the antibody platform used for immunohistochemistry (IHC) nor participation in round robin tests of local pathology institutes correlated with the HER2 test deviation rate. In contrast, we found that tumor characteristics such as primary tumor location and phenotype had an impact on test deviations: more HER2 test deviations were seen in distal GC compared to EGJC as well as in the diffuse versus intestinal subtype according to Laurén’s classification.

HER2 heterogeneity is common in GC, even in early stages (Kanayama et al. 2018). There is no generally agreed definition for HER2 heterogeneity in gastric cancer. Some authors use the relative number of HER2 + stained tumor cells, but thresholds vary from < 30% (van Cutsem et al. 2015), < 40% (Haffner et al. 2021), to < 60% (Kanayama et al. 2018; Wang et al. 2014) or even < 100% tumor cells stained HER2-positive (Wakatsuki et al. 2018; Yagi et al. 2019). Other authors determined HER2 heterogeneity by deviating HER2 status in a set of primary biopsies (Kaito et al. 2019), of resection specimen (Xu et al. 2019) or in paired specimens from the primary tumor and metastasis (Peng et al. 2015). Although HER2 heterogeneity in gastric cancer has no commonly agreed definition, it is associated with limited trastuzumab benefit and decreased overall survival in trastuzumab-treated patients, as was suggested by several groups (Haffner et al. 2021; Kaito et al. 2019; Wakatsuki et al. 2018). We conclude that for distal gastric cancer location and for the diffuse subtype where HER2 positivity in general is less common (Baretton et al. 2019; He et al. 2015; Huang et al. 2013; Koopman et al. 2015; van Cutsem et al. 2015; Yoon et al. 2012; Barros-Silva et al. 2009; Cappellesso et al. 2015; Cho et al. 2013; Chua and Merrett 2012; Gomez-Martin et al. 2013; Gómez-Martin et al. 2012; Grabsch et al. 2010) weak HER2 expression and intratumoral heterogeneity account for more deviating test results.

Strengths of the presented investigation are the reporting of the centrally performed standardized HER2 tests by both IHC and ISH for all received specimens. Furthermore, we established a large prospective multicenter cohort with valid clinical data in a real-world setting.

Nevertheless, a limitation of our study might be the central use of the HER2-antibody CB11 for IHC, which is not as widely used as the 4B5 clone and the HercepTest. Although less sensitivity was seen for CB11, this clone presented the highest specificity compared to HercepTest and others (Asioli et al. 2012; Cappellesso et al. 2015; Cho et al. 2012; Grillo et al. 2013). In our own study, test results obtained by CB11 were associated with a survival advantage in trastuzumab-treated patients (Haffner et al. 2021). In addition, high concordance between ISH and IHC with CB11 was seen in the VARIANZ (98.3%) and other studies (Barros-Silva et al. 2009; Cho et al. 2012). Nevertheless, for further examination of local and central HER2 determination, the use of a second antibody should be considered as well as application of an automatized scoring system might be considered. Another limitation is that a significant number of local laboratories provided neither specification of the applied antibodies nor information on participation in quality assurance programs such as round robin tests. Finally, central HER2 determination was not necessarily performed on the same tumor block as it was done in local laboratories which adds to the complexity of achieving concordant diagnosis.

Deviating central and local HER2 reports were already reported for various gastric cancer cohorts (Byeon et al. 2017; Huemer et al. 2020; Monges-Ranchin et al. 2016; Press et al. 2017). Discordant results are particularly often found in patients tested locally HER2 + (Haffner et al. 2021; Huemer et al. 2020; Janjigian et al. 2020). Patients with centrally confirmed HER2 + status treated with trastuzumab showed better survival outcomes (Haffner et al. 2021; Huemer et al. 2020). We conclude that a central confirmation of the HER2 status is a correlate of lower HER2 heterogeneity which is an indicator for better treatment efficacy with an anti-HER2-targeted drug such as trastuzumab.

Identification of HER2 + in gastric and EGJ cancer patients remains an important step in the management of patients with advanced GC and EGJC, but is still challenging. Adaption of HER2 thresholds to identify patients benefiting from trastuzumab treatment has been already postulated (Gomez-Martin et al. 2013; Haffner et al. 2021). Our data suggest that only patients with low HER2 heterogeneity may benefit from trastuzumab treatment. Selection of patients with low HER2 heterogeneity, defined as confirmed HER2 + status in paired specimen, might improve survival outcomes with targeted treatment, prevent overtreatment and associated side effects and costs, and may enable successful studies of other promising HER2-targeting drugs.

## Supplementary Information

Below is the link to the electronic supplementary material.

Supplementary file1 (BMP 46873 KB)

Supplementary file2 (DOCX 66 KB)

## Data Availability

Not applicable.
