# Supplementary figures and images for: Deviating HER2 test results in gastric cancer: analysis from the prospective multicenter VARIANZ study
Source: J Cancer Res Clin Oncol. 2022 Aug 27;149(3):1319–29. doi: 10.1007/s00432-022-04208-6 (PMC9984518; doi:10.1007/s00432-022-04208-6)

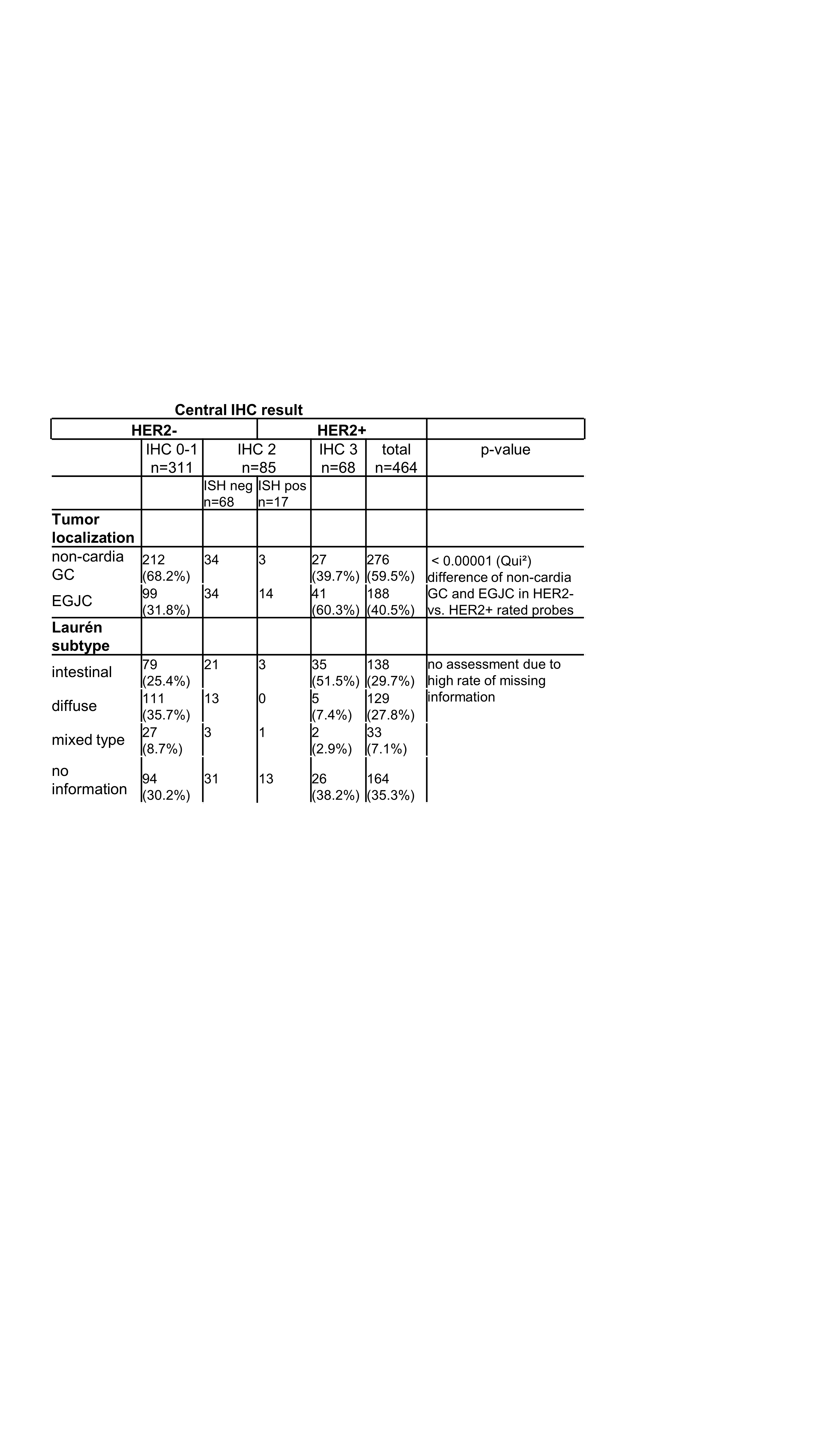

Supplement: Supplementary file 1 — Supplementary file1 (BMP 46873 KB) [file 432_2022_4208_MOESM1_ESM.bmp]
