# Supplementary material for: Deviating HER2 test results in gastric cancer: analysis from the prospective multicenter VARIANZ study
Source: J Cancer Res Clin Oncol. 2022 Aug 27;149(3):1319–29. doi: 10.1007/s00432-022-04208-6 (PMC9984518; doi:10.1007/s00432-022-04208-6)
Supplement: Supplementary file 2 — Supplementary file2 (DOCX 66 KB) [file 432_2022_4208_MOESM2_ESM.docx]

## Analyzed samples in fig. 2 (n = 340)

**registered (n = 548)**

**Central HER2 tests (n = 521)**

**Central and local HER2 result (n = 404)**

Excluded (n = 27)

- no tissue was available (n = 19)
- no tumor tissue was left on the specimen (n = 8)

Excluded (n = 117)

- HER2 was not tested in local pathology (n = 84)
- local pathology is equivalent to central pathology (n = 33)

Excluded for figure 2

- local HER2 test result known, but unknown IHC score (n = 64)

**Local HER2 +**

**Local HER2 -**

- no central ISH result (n = 27)

Local IHC 0/1
(n=178)

Local IHC 3+
(n=124)

Local IHC 2+
(n= 28) (n=10)

## With central ISH result (n = 313)

Local IHC 2+
(n= 25) (n=10)

Local IHC 0/1
(n=166)

Local IHC 3+
(n=112)

## Central Confirmation of local HER2 test result (n = 313)

28/28 5/10
(100%) (50%)

57/124
(46%)

173/178 (97.2%)

Lost to follow-up (give reasons) (n= )

Discontinued intervention (give reasons) (n= )
